# Impact of gut microbiome on skin health: gut-skin axis observed through the lenses of therapeutics and skin diseases

**DOI:** 10.1080/19490976.2022.2096995

**Published:** 2022-07-22

**Authors:** Md. Rayhan Mahmud, Sharmin Akter, Sanjida Khanam Tamanna, Lincon Mazumder, Israt Zahan Esti, Sanchita Banerjee, Sumona Akter, Md. Rakibul Hasan, Mrityunjoy Acharjee, Md. Sajjad Hossain, Anna Maria Pirttilä

**Affiliations:** a Department of Production Animal Medicine, Faculty of Veterinary Medicine, University of Helsinki, Helsinki, Finland; b Department of Microbiology, Jagannath University, Dhaka, Bangladesh; c Department of Bioscience, Graduate School of Science and Technology, Shizuoka University, Shizuoka, Japan; d Institute of Biology, Eötvös Loránd University, Budapest, Hungary; e Ecology and Genetics, Faculty of Science, University of Oulu, Oulu, Finland

**Keywords:** Gut microbiome, gastrointestinal health, gut dysbiosis, skin disease, probiotics, prebiotics, skin-gut axis, dietary components

## Abstract

The human intestine hosts diverse microbial communities that play a significant role in maintaining gut-skin homeostasis. When the relationship between gut microbiome and the immune system is impaired, subsequent effects can be triggered on the skin, potentially promoting the development of skin diseases. The mechanisms through which the gut microbiome affects skin health are still unclear. Enhancing our understanding on the connection between skin and gut microbiome is needed to find novel ways to treat human skin disorders. In this review, we systematically evaluate current data regarding microbial ecology of healthy skin and gut, diet, pre- and probiotics, and antibiotics, on gut microbiome and their effects on skin health. We discuss potential mechanisms of the gut-skin axis and the link between the gut and skin-associated diseases, such as psoriasis, atopic dermatitis, acne vulgaris, rosacea, alopecia areata, and hidradenitis suppurativa. This review will increase our understanding of the impacts of gut microbiome on skin conditions to aid in finding new medications for skin-associated diseases.

## Introduction

Skin and gut both are active, complex immunological and neuroendocrine organs that are exposed to the outside environment on a frequent basis and host a wide range of microbiomes.^1,2^ The skin and gut must operate appropriately in order to enable organisms to maintain homeostasis and survive.^3^ Notably, the skin is the body’s largest organ and serves as a defensive obstruction against injuries and microbial assault.^4^ The gut, on the other hand, consists of trillions of microbial communities, being recognized as a virtual organ closely associated with health and longevity of the host. The gut microbiome has both beneficial and adverse impacts on normal physiology and homeostasis of both gut and skin tissues.^3,5^

The three most essential roles that the gut microbiome plays from birth are protection, providing metabolic activities and immune system development and regulation.^6,7^ At the beginning of life, gut microbial communities have a role in defending the host against pathogenic organisms. Throughout the life, they provide metabolic services, such as digestion of breast milk and other food. Members of microbiome help in degradation of toxins and drugs and in the biosynthesis of vitamins.^6^ The gut is considered to be in a state of symbiosis, because it is populated by a diverse group of microorganisms, and the host tolerates these commensal bacteria and associated benign antigens.^8–10^ The ability of the immune system to build a tolerance to benign antigens is built through ontogeny and the reduction of microbiome-dependent inflammatory responses.^10,11^ For example, in mice, long-term colonization by benign *Hymenolepis diminuta* results in modification of the immune system without causing dysbiosis and give protection against immune-mediated inflammatory diseases (IMIDs).^12^

However, any alteration among gut microbial diversity (dysbiosis) can increase host vulnerability and disrupt mucosal immunological tolerance,^7^ which can subsequently influence skin health.^13^ Several dermatologic conditions, such as acne, atopic dermatitis, psoriasis, and rosacea are linked with intestinal dysbiosis.^223^ Many studies have associated gastrointestinal health with skin homeostasis and allostasis, and there is evidence of a bidirectional interaction between the gut and the skin.^2^ Members of the gut microbiome can influence skin conditions through their metabolic activity and immunological impact.^3^ For example, commensal gut microbes can foster skin allostasis by controlling T-cell differentiation.^2^

Based on existing studies, we hypothesize that the gut microbiomes are vital to the gut-skin axis.^3^ Thus, this review is intended to evaluate the interlinks between gut and skin microbiome and following skin conditions. Our primary objective is to review and analyze microbial ecology of skin and gut tissues and to study the characteristics and mechanisms of microbe-transmitted interactions along the gut-skin axis. Diet and probiotics have an enormous impact on the composition and metabolic activities of gut microbiome, which subsequently impacts the skin.^14^ Earlier, Polkowska-Pruszyska et al.,^15^ have discussed how changes in gut microbial communities could trigger an immunological response, resulting in allergies, acne vulgaris, atopic dermatitis (AD), alopecia areata (AA), rosacea, hidradenitis suppurativa (HS) and other skin diseases. De Pessemier et al.,^16^ recently demonstrated an association between dietary products and skin dysbiosis in their research. The present review paper, on the contrary, will give an integrated and structured view of the therapeutic effects of drugs, prebiotics, and probiotics on the gut microbiome and skin, as well as dietary effects on skin conditions. The secondary objective of this paper is to discuss the connection between microbial communities of the gut and skin, and their significance in dermatologic conditions, such as psoriasis, atopic dermatitis (AD), acne vulgaris, rosacea, alopecia areata (AA), and hidradenitis suppurativa (HS).

## Methodology

### Criteria for literature search, study selection, and exclusion

**Figure 1.**
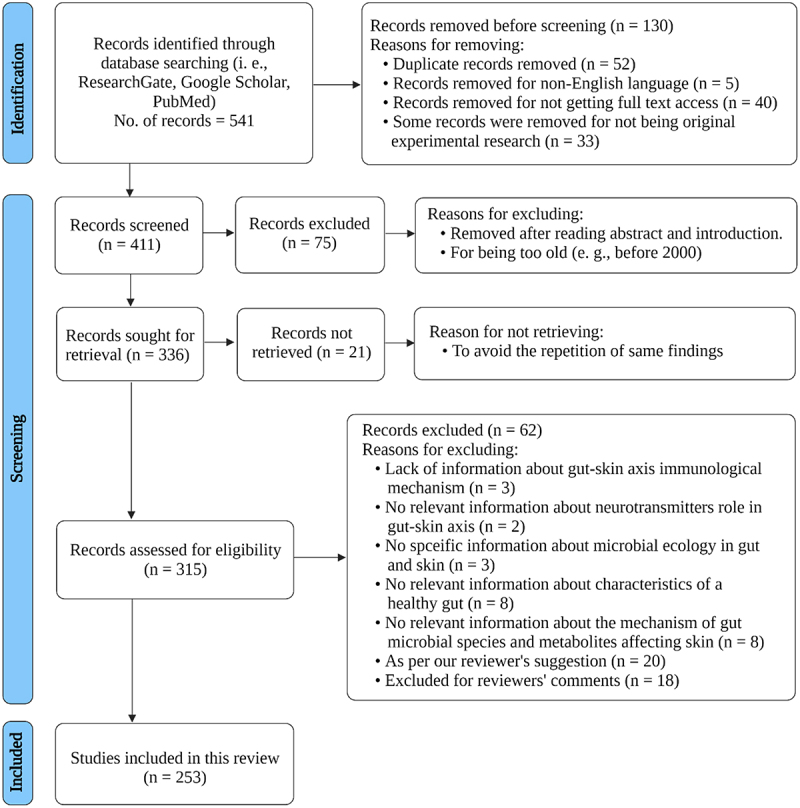
**Prisma flowchart.** This diagram represents the Prisma flowchart and demonstrates database searching, screening, excluding, retrieval, and eligibility findings for the final full-text studies used in this review. This illustration is based on^17^ and created with BioRender.com (2022).

The present review paper followed the PRISMA guideline. Figure 1. depicts a flow diagram. Articles published between 2000 and 2021 were searched to find related information on the association between the gut microbiome and skin health. Articles were searched on Google Scholar, ResearchGate and PubMed using keywords like gut microbiome, skin microbiome, intestinal dysbiosis, skin–gut interactions, gut–skin microbiome, skin homeostasis, skin–gut allostasis, commensal bacteria, alteration in the gut microbiome, skin and gut microbial ecology, the role of the gut microbiome in the pathogenesis of skin diseases, gut microbes in skin disease prevention and treatment, gut–skin axis, diet influencing skin inflammation through gut microbes, drugs affecting gut-skin axis, biologics influencing skin-gut axis. The selection of such keywords ensured that all papers relating to gut microbiota and skin diseases were included. The current analysis only included peer-reviewed, high-quality research data, omitting preprints, non-English papers, and duplicate databases.

### The skin and gut microbial ecology

As the composition of human microbiome varies significantly between individuals and contributes both positively and negatively to host immunity, a broad understanding on the microbial ecology of humans is necessary. Humans acquire a maternal microbiome mainly from the birth canal, and the microbial community changes over time.^18,19^ Skin, oral tissues, airways, gut and vagina generally harbor hundreds of microbial genera and species unlike other tissues and organs.^18,20^

The skin is the largest organ of the human body and provides multiple niches for colonization by diverse microbial communities, such as the stratum corneum, hair follicles, and sebaceous glands.^21^ Skin acts as the boundary between the external and internal environment of the human body, and the skin microbiome is an interface that contributes to human immunity.^22^ Culture-independent methods have revealed that healthy human skin harbors >1,000 bacterial species, mainly within the genera *Brevibacterium, Propionibacterium, Micrococcus, Staphylococcus, Streptococcus*, and *Corynebacterium*, and *Malassezia* is the main fungal genus.^21,23^ The normal microbial communities of the skin can interact with the host in both commensal and parasitic ways.^24^

**Figure 2.**
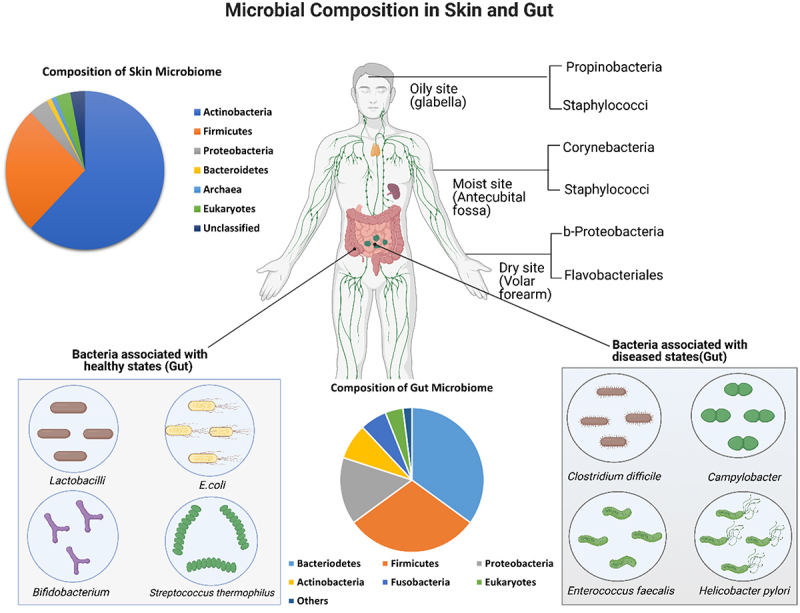
**Microbial composition of gut and skin**. Skin, the largest organ of the human body, shelters numerous commensal microbes (bacteria, fungi and viruses) and prevents entry by foreign pathogens by acting as a physical barrier. Skin can be broadly categorized as sebaceous or oily (glabella), moist (antecubital fossa) or dry (volar forearm), according to the physiological characteristics of each skin site. Specific microbial groups dominate different skin sites. Like skin, human gut is a home to innumerable amounts of microbes. A number of gut bacteria (e.g, *Lactobacilli, E. coli, Bifidobacterium, Streptococcus thermophilus*) contribute to maintenance of human health state, whereas others (e.g., *Clostridium difficile, Campylobacter, Enterococcus faecalis, Helicobacter pylori*) are more prevalent in disease states. The illustration was adapted from “Immune Organs in the Human Body”, by BioRender.com (2022). Retrieved from https://app.biorender.com/biorender-templates, associated information based on .^25–27,28^

A number of studies have highlighted the predominant factors contributing to the ecosystem of the human skin microbiome. Several skin regions with versatile characteristics, such as dry sites (volar forearm, buttock, hypothenar palm), moist sites (axillary vault, antecubital fossa, inguinal crease, umbilicus), and oily sites (glabella, alar crease, occiput, manubrium) differ from each other based on microbial composition (Figure 2).^29^

Ecology of gut microbes has been extensively studied for decades, as the human gastrointestinal (GI) tract is one of the most complex systems in the human body. The gastrointestinal system starts from the oral cavity and finishes in the anus after passing through the stomach and intestines.^30^ The GI tract is considered more microbially enriched than any other organ in the human body and has the highest diversity of microorganisms. Up to 10^13^ microbial cells, belonging to all three domains of life (Bacteria, Archaea and Eukarya), and viruses, comprise the microbial community of the GI tract. Microbial diversity in the gut varies between individuals.^18,31^ Based on chemical composition and physical state, the GI tract can be divided into distinct sections. The upper portion of the GI tract, stomach, and small intestine have comparatively low numbers of bacteria (10^3^ to 10^4^ cells in total) due to low pH and shorter transition period. The colon is the most colonized region of the GI tract, where an estimate of 10^10^ to 10^11^ bacterial cells resides.^30^ The gut can harbor 1000 different bacterial species belonging to phyla such as *Bacteroidetes, Firmicutes, Actinobacteria, Proteobacteria*, Verrucomicrobia, Fusobacteria, Tenericutes, Spirochaetes , Cyanobacteria, and Saccharibacteria (Figure 2).^30–32^

Fungal diversity, in contrast, is relatively limited in the human gut. Pyrosequencing has revealed nearly 66 genera of fungi from 98 human individuals; the prevalent fungal genera being *Saccharomyces, Candida*, and *Cladosporium*.^33^ Abundance of fungi such as *Candida, Aspergillus, Fusarium*, and *Cryptococcus* in the gut may have a pathogenic effect on the host.^30^ For example, children with type I diabetes have high numbers of *Saccharomyces* and *Candida* yeasts in their gut.^34^ Among Archaea, the predominant genera in the human gut are *Methanobrevibacter, Methanosphaera, Nitrososphaera, Thermogynomonas*, and *Thermoplasma*.^33,35^ Viruses and phages, on the other hand, can act as reservoirs of genetic material in the gut and destroy microbial cells.^36^

### Characteristics of a healthy gut

Microbes colonize the human gut at birth.^37–40^ The initial colonization plays an essential role in determining the composition of the gut microbiome as the person grows older.^41^ Progression of the intestinal microbiome is influenced by contact between host and microbes. For instance, analogous microbiomes are present in the intestine of infants and the vagina of their mothers.^42^ During the early life (above the age of 3), the composition of gut microbiome changes with time until it becomes relatively stable.^5^ A normal gut harbors bacterial genus such as *Bacillus, Lactobacillus, Enterococcus, Clostridium*, and *Ruminococcus*.^43^ One of the most notable changes in the gut microbiome is the ratio between the phyla Firmicutes and Bacteroidetes, as higher Firmicutes level is reported in obesity.^44^ The gut microbiome has a number of functions, including gathering of indigestible food particles in feces and assimilating nutritious particles, such as vitamins and minerals,^45^ while collaborating with the liver to detoxify and eliminate xenobiotics, which are toxic foreign compounds that are omnipresent in the environment.^46^ Millions of microbial genes have been identified in the gut that support essential and necessary human functions.^47,48^ The gut is the primary site of intricate interactions between the genes and the extrinsic immunological influence on the body, making it one of the most important organs for communicating with the environment.^49^ Several microbial ecosystems consisting of the entire mucosal lining are maintained in the human gastrointestinal tract.^50^ When villi, microvilli, crypts, and folds are considered, the gut epithelium of an adult spans a surface area of around 300 m^2^; this barrier typically contains just one cell layer and is therefore vulnerable. Bacteria that enter through the gut wall and into the bloodstream can cause a systemic inflammation in the absence of a healthy intestinal wall.^51^ The gut wall, on the other hand, is protected by a variety of chemical and physical innate defense mechanisms that function in tandem with a local adaptive immune system.^52^

The role of gut microbiome in epithelial cell renewal and intestinal integrity regulation is crucial to a person’s overall and intestinal immune systems.^51^ The local adaptive immune system is dominated by an immunoglobulin A (IgA)-producing B-cell population, which primarily offers an anti-inflammatory first-line response by producing secretory IgA (sIgA) antibodies that conduct immune exclusion.^52,53^ Immune tolerance toward dietary and environmental antigens is modified by gut microorganisms, which also protect against potential pathogens.^2^ Such protection is achieved, besides directly triggering the immune protective responses, indirectly by attachment of microbes to epithelial cells.^48^ Their attachment to gut epithelium can provide colonization resistance in the gut. By such process, resident microorganisms can block colonization of the gut by exogenous pathogenic microbes, such as *Clostridium difficile* and *Helicobacter pylori*.^54^

### Gut-skin communication through immuno-cross-linking

**Figure 3.**
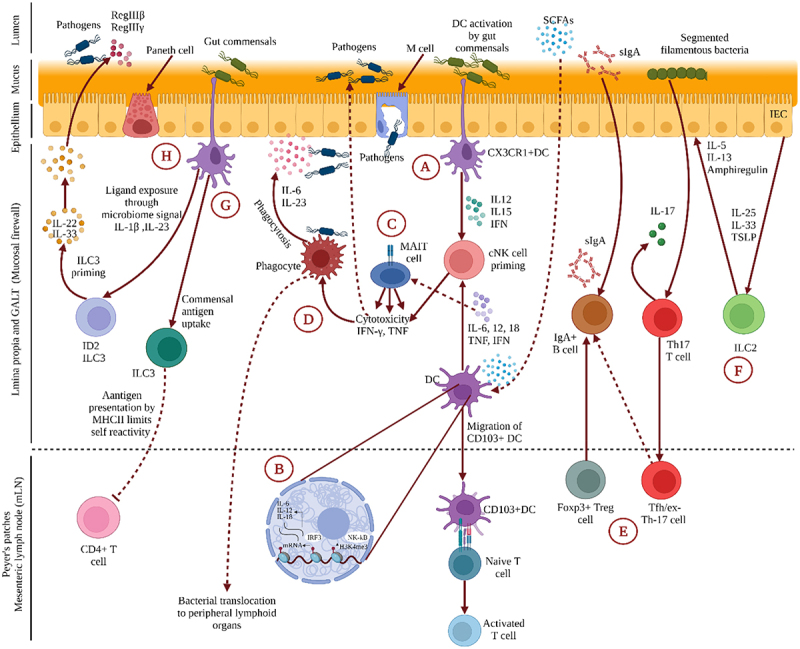
**Gut-Skin communication through immuno-cross-linking.** This illustration represents the immunological crosstalk between the gut and skin. **(A)** CX3CR1+ DCs generate dendrites for phagocytosis at homeostatic condition, whereas CD103+ DCs relocate to Peyer’s patches or mesenteric lymph nodes to deliver antigens to naive T lymphocytes. DC secretes interleukin (IL)-12, IL-15, and interferon (IFN) in response to commensal activation to stimulate conventional NK (cNK) cells. **(B)** As metabolic by-products, short-chain fatty acids (SCFAs) upregulate H3K4me3 in DC and enhance the production of IL-6, IL-12, IFN, and tumor necrosis factor (TNF), which is an alternative way to train cNK cells. Trained cNK cells have the necessary cytotoxicity and cytokine production capacity to fight bacteria and viruses. **(C)** MAIT cells can be directly stimulated to create IFN-γ by IL-12 or IL-15 in combination with IL-18 produced by APCs in response to TLR ligands. **(D)** TNF-like protein, a gut-associated pro-inflammatory cytokine, activates MAIT cells when coupled with IL-12 and IL-18. Phagocytes help the body defend itself by phagocytosing and producing cytokines like IL-6 and IL-23. **(E)** Foxp3+ Treg cells and Tfh/ex-Th17 cells cluster in Peyer’s patches, promoting B cell class switching and secretory (s)IgA production. These help to compartmentalize the commensal microbiome and modulate the diversity of the homeostatic microbiome. **(F)** ILC2 is activated by IL-25, IL-33, and thymic stromal lymphopoietin (TSLP) produced by intestinal epithelial cells (IEC) in response to commensal bacteria. **(G)** ILC3 expressing MHC II is capable of delivering commensal antigens to CD4 + T cells, reducing their self-reactivity. **(H)** In an ID2-dependent manner, microbial signals are also used to prime ILC3. ILC3, which has been primed secretes IL-22 and participates in the pathogen defense by stimulating the synthesis of antimicrobial peptides, such as REGIIIβ and REGIIIγ. This illustration was based on ^55–58^ and created in BioRender.com (2022).

Gut-skin communication occurs through the activities of immunological components which are present between the gut and the skin. Regulating host’s interaction with the microbiota is a fundamental function of immune system, thereby the regions colonized by commensals, such as the skin and the GI tract, encase the substantial volume of immune cells in the body. Through the dominant activity on the immune system, the commensal microbial communities play an important role in boosting barrier immunity along with their own confinement to safeguard their ecological niche. Curtailing the contact between microorganisms and the gut epithelial membrane to minimize the inflammatory responses and microbial translocation is crucial in preserving the host’s homeostatic balance. To achieve this segregation, gut epithelial cell barrier, mucus layer, T cells, IgA, and dendritic cells (DCs) collectively forge the shield named ‘mucosal firewall’ (Figure 3). The mucosal firewall limits the translocation of commensal bacteria to the lymphoid tissues, preventing the inflammation of the gut and skin.^59^ These lymphoid tissues are usually known as gut-associated lymphoid tissues (GALTs) (Figure 3). GALTs are typically mucosa-associated lymphoid tissues (MALTs) that work as a barrier between the host and the environment.^55^ GALTs are comprised of microfold cells (M cells), which have evolved into phagocytose and transcytose (a process through which particles are transported across the mucosal barrier to the lamina propria via unique cellular pathways) gut lumen macromolecules, particulate antigens, and harmful or commensal bacteria through epithelium (Figure 3).^60,61^ Along with M cells, conventional lymphocytes (regulatory T cells (Tregs), helper T cells (Th cells), cytotoxic T lymphocytes, IgA producing B cells), and professional phagocytes (DCs, mast cells, neutrophils, and macrophages) and unconventional lymphocytes, such as innate lymphoid cells (ILCs) and mucosal-associated invariant T (MAIT) cells, are also the constituents of GALTs (Figure 3).^55,62^ In addition, reports indicate that gut microbiota is behind the fundamental development mechanism of GALTs (Figure 3).^63,64^ Peyer’s patches, crypt cells of the intestinal epithelium, isolated lymphoid follicles (ILFs) of the intestine, appendix, and mesenteric lymph nodes (mLNs) are the most common histologic components of GALTs.^63,64^ The hematopoietic cell type lymphoid tissue inducer (LTi) cell and its interplay with gut microbial colonization control the formation of gut secondary lymphoid organs.^55^

Both conventional lymphocytes and professional phagocytes secrete antimicrobial peptides (AMPs) for the maintenance of homeostasis. AMPs are convoluted evolving compounds produced by gut epithelial cells, paneth cells, and immunological cells in the digestive tract (Figure 3).^65^ AMPs, such as α- and β- defensins, are secreted by localized immune cell types, namely macrophages, T cells, B cells and mast cells (MC).^66^ Furthermore, MCs can produce the AMP cathelicidin and contribute to microbiome-tissue homeostasis in the dermis. Pathogens or their components can directly bind to the TLRs, (NOD)-like receptors (NLRs), and (RIG-I)-like receptors (RLRs) and activate complement receptors of MCs, subsequently releasing inflammatory mediators, which aid in antimicrobial immune responses.^67^ By expression of co-stimulatory molecules and secretion of inflammatory cytokines, TLR4 elicits innate responses. An earlier study in a mouse model substantiated that TLRs generally function as pattern-recognition receptors (PRRs) with the ability to recognize a wide range of pathogen-associated molecular patterns (PAMPs), including proteins, lipoproteins, lipids, nucleic acids, and glycans, and aid the initiation of innate immunity.^68^ Development of ILFs occurs as soon as PRRs recognize PAMPs of enteric bacteria and activate the downstream signaling pathways. Several AMPs, such as REGIIIβ and REGIIIγ can be produced as the result of Peyer’s patch priming by TLR pathways. On the other hand, enteric bacterial infection can occur due to the inhibition of TLR pathway. For example, TLR2 deficiency impairs the integrity of the intestinal epithelial barrier and disrupts the balance between commensal bacteria and host defense, exacerbating colitis.^55,69^

As antigen presenting cells, DCs play an important role in producing tight junction proteins and extending dendrites into the lumen through the junctions.^70^ Through the adherence to C-X3-C Motif Chemokine Receptor 1 (CX3CR1), DCs give rise to the formation of trans-epithelial dendrites and the delivery of antigens for sampling (Figure 3). DCs are also capable of forming trans-epithelial dendrites and phagocytosing invasive enteric pathogens.^71^

ILCs play imperative parts in immunity, homeostasis, and inflammation in various tissues, including the intestine, lungs, skin, liver, adipose tissue, and mesenteric lymph nodes.^72,73^ ILCs are classified in three groups by the developmental process, transcription factor expression, and secretion profiles of interleukins (IL)-17A, IL-17 F, IL-22, and GM-CSF, namely, ILC1, ILC2, and ILC3, which are the innate counterparts of T helper cells, Th1, Th2, and Th17, respectively.^73–75^ Among the helper ILC subsets, ILC1 has a comparatively low frequency in the fetal intestine, as the gut microbiome is not established. This indicates that ILC1 development depends on commensal bacteria.^55,71^ Th1 cells and ILC1s eradicate viruses, bacteria, or protozoa from the body through production of IFN-γ.^75^ Th2 and ILC2 cells, which produce IL-5 and IL-13, respectively, are behind development of allergies and assist in the elimination of helminths. ILC3s and Th17 cells also contribute to autoimmunity by the secretion of IL-17 and IL-22, respectively, which provide protection against fungal and extracellular bacterial infections (Figure 3).^75^

Meanwhile, the most common subgroup of T-cells that can identify bacterial particles is mucosal-associated invariant T (MAIT) cells, also known as evolutionarily conserved T-cells.^76^ MAIT cells play a crucial role in elimination of bacterial infections from the body, and their role in defense against viral infection has also been reported.^67^ MAIT cells detect antigens via Major histocompatibility complex class I-related gene protein (MR1), a protein predominantly expressed by B cells.^77^ When MAIT cells come into contact with a variety of bacteria, they respond by detecting vitamin B2 biosynthesis pathway products via T-cell receptor (TCR) recognition.^67^

### Mechanisms of the interaction at the gut-skin axis

**Figure 4.**
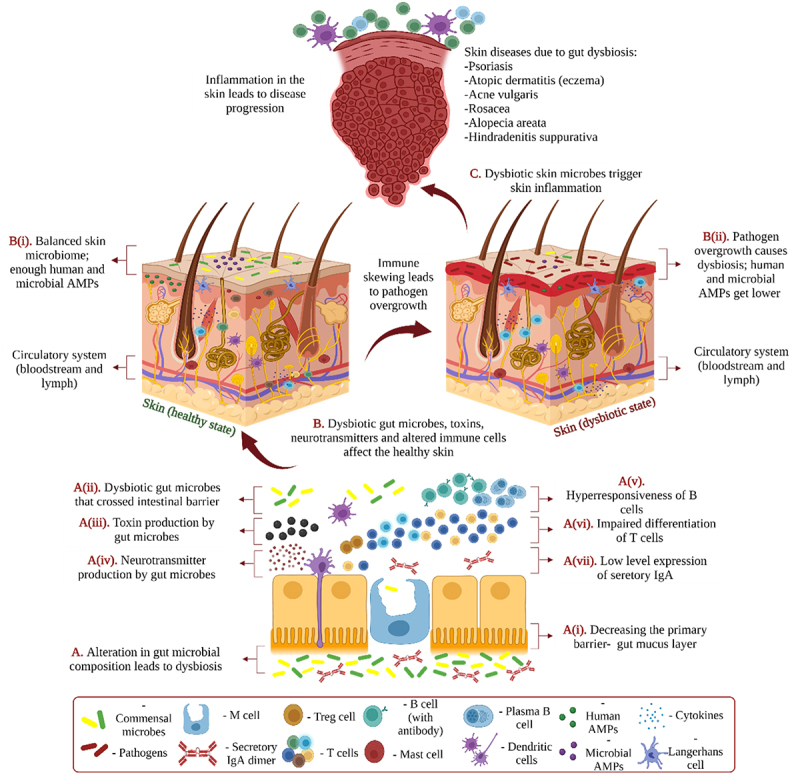
**Mechanisms of the interaction at the gut-skin axis.** This illustration represents the underlying mechanisms of gut-skin interaction. Various dietary components, illnesses, lifestyles, prebiotics, antibiotics, probiotics, and novel biological drugs can alter gut microbial communities. **A**. The alteration can lead to dysbiosis, which can further **(i)** decrease the gut mucus layer, **(ii)** results in the passage of microbes through the intestinal barrier, **(iii)** cause the production of toxic products, **(iv)** induce harmful effects by neurotransmitters of the gut microbes or the host, **(v)** produce B cell hyperresponsiveness, **(vi)** impair T cell differentiation, **(vii)** create low levels of IgA secretion. **B**. Dysbiotic gut microbes, toxic products, neurotransmitters, and altered immune cells pass through the circulatory system turning the skin condition from healthy (**left**) to dysbiotic (**right**). **(i)** Healthy skin possesses a balanced composition of microbes and proper quantities of human and microbial AMPs. **(ii)** A dysbiotic skin condition is induced by the pathogen due to improper immune system functioning and low quantities of human and microbial AMPs. **C**. Dysbiotic skin microbes trigger skin inflammation and can be involved in the onset of a variety of skin illnesses. This illustration is based on ^15^and ^16^and created with BioRender.com (2022).

Disruption of gut integrity, and an imbalance within microbial communities can have a significant impact on the overall homeostasis of skin.^2^ Gut-skin axis is a term used for the intricate interaction between the gut and the skin.^16^ The gut microbiome interacts with the skin largely to manage systemic and local inflammation through engaging with the immune system (Figure 4).^15,78^ The microbial communities maintain the gut barrier integrity mainly by converting undigestible complex polysaccharides into vitamins (specifically K and B12), and SCFAs (specifically butyrate and propionate).^79,80^ For example, butyrate wanes the intestinal barrier permeability and enhances epithelial barrier integrity.^16^ The mucus layer of the gut acts as the primary barrier and prevents microbial relocation to other host tissues (Figure 4).^81,82^ The gut mucosal defense is provided by innate immune cells of GALT. They recognize nonspecific infections and activate both the innate and adaptive immune systems by presenting those antigens.^63,83^ AMPs, macrophages, and CD103^+^ CD11b^+^ DCs mainly limit the translocation of pathogenic microbes by eliminating them.^81,84,85^ Defensins act against bacteria by generating pores in their membranes. This results in cell death if appropriate thresholds are exceeded. Cathelicidins (LL-37 in humans) help to keep the epithelial barrier intact. While their principal mechanism of action is to break bacterial membranes, they also possess immunomodulatory effects. Enhanced tight junction protein production, as well as post-translational effects, such as tight junction repositioning, are principally responsible for the gut epithelial barrier integrity maintenance. As a result, cathelicidins predominantly are employed when the epithelial barrier is breached.^65^ Differentiation of gut commensal bacteria-specific Tregs, IgA-producing B cells, and Th17 cells are the outcomes of commensal antigens presentation by DCs.^84^ DCs establish the specificity of CD4+ Th17 cells toward commensal microbes as the result of Major histocompatibility complex II (MHCII) antigen presentation. The CD4+ Th17 cells produce the cytokine Interleukin 22 (IL-22), which enhances secretion of host AMPs.^86,87^ The integrity of the intestinal barrier along with the action of mucus, immune cells, IgA, and antimicrobial peptides (AMPs) produced by epithelial cells prevents the entrance of gut bacteria into the bloodstream, ultimately maintaining skin homeostasis.^59^ Especially, secretory IgA controls the inflammatory responses against the gut microbes by spatially dissociating the host tissue and gut microbes.^11,88,89^ The commensal bacteria-specific lymphocytes accumulate in Peyer’s patches and lamina propria of the gut, which shapes the gut microbial profile toward homeostatic balance. Promotion of class switching by Tregs and IgA production against the commensal bacteria takes place in the Peyer’s patches of the gut.^90,91^ However, the link between skin health and immunological responses caused by the gut microbiome is still largely unclear and requires further research.^92,93^

In the first part of the twentieth century, dermatologists Stokes and Pillsbury were the first to suggest that the gut and skin communicate with the brain.^3,94^ GABA, acetylcholine, dopamine, and serotonin are among the neurotransmitters produced by gut microbes. These neurotransmitters can modulate the function of the skin through the nervous system. They can also create systemic effects by entering the bloodstream through the intestinal epithelium (Figure 4).^95^ For example, an experiment conducted on NC/Nga mice (an inbred mouse line employed as a human AD model) demonstrated the role of GABA in AD-like skin lesion mitigation. This experiment led to the conclusion that by increasing the production of serum immunoglobulin E (IgE) and splenocyte IL-4, GABA balances the T helper cell type 1 (Th1) and T helper cell type 2 (Th2) levels, keeping the Th1 predominant, which effectively wanes AD-like skin lesions in human.^96^ By suppressing the type I collagen degrading enzyme matrix metalloproteinase-I (MMP-I), GABA also increases the expression of human type I collagen (COL1A1 and COL1A2) and maintains skin elasticity.^97^ Neurotransmitters can also create negative effects, e.g., dopamine can inhibit hair growth by stimulating catagen induction.^98^

Antibiotics, prebiotics, probiotics, lifestyles, long-term diets, and illnesses can influence the gut microbiome. Furthermore, changes in the major strains of the gut microbiome can occur as people age.^99–102^ Skin inflammation may also result from minute changes in a single bacterial species of the intestinal microbiome.^92,93^ These, in turn, may lead to diseases e.g., acne, alopecia areata, atopic dermatitis, psoriasis, rosacea, and hidradenitis suppurativa (Figure 4).^16,103,104^ Table 1 lists the microbial species and metabolites associated with various skin effects.

**Table 1.** Microbial species and metabolites from the gut that have been associated with skin effects.

**A. Microbial species**					
Organism		Effects on skin	Mechanism		References
*Faecalibacterium prausnitzii, Akkermansia muciniphila* and *Ruminoccocus*		Protection against psoriasis	Prevention of colonization of pathogenic flora on skin by competitive inhibition and the SCFAs production		^158,159,169,213^
*Helicobacter pylori*		Rosacea-related signs and symptoms	Production of cytotoxin and by proliferating the production of reactive oxygen species-nitric oxide [NO], which causes gut mucosal inflammation and changes physiological processes in the skin including vasodilation, inflammation and immunomodulation.		^194^
*Faecalibacterium prausnitzii*		Chronic atopic dermatitis progression resulting in gut epithelial barrier impairment	Dysregulation of gut epithelial inflammation		^180^
*Lactobacillus casei*		Decrease skin inflammation	Alteration of the number of cytotoxic CD8 + T cells		^171^
*Lactobacillus paracasei*		Reduce the size of acne lesions as well as inflammation	Inhibition of mast cell degranulation, TNF-α release, edema and vasodilation, and thereby speeding up the restoration of barrier function		^145,171^ ^136^
*Bifidobacterium animalis* subsp. *lactis* [LKM512]		Reduce the scratching behavior in atopic dermatitis	Increase of levels of the kynurenic acid metabolite		^223^
*Bacteroides thetaiotaomicron*		Alleviate the allergic symptoms of atopic dermatitis as well as Crohn’s disease like other chronic inflammatory diseases	Anti-inflammatory action		^228^
Larger number of *Clostridium difficile* and *Escherichia coli*		Onset of atopic dermatitis symptoms in childhood	Immune dysregulation as a result of decreased Treg cell inducing beneficial bacteria		^16,224^
Decrease in Firmicutes and increase in Bacteroides		Development of acne vulgaris	Dysbiosis by altering the serological cytokine levels promoting inflammation		^15,188^
**B. Metabolites**					
Metabolites	Effects on skin		Mechanism	References	
SCFAs	Increase the epithelial barrier function and skin-inflammation		Development of Tregs within the colon, DCs precursors, and IL-10 production	^120,229^ ^13,106^	
GABA	Itch restriction		Inhibition of neurons which are responsible for itch-signaling in the spinal cord	^230,223^	
Tryptophan	Regulate skin inflammation		Activation of AhR and inhibition of TSLP production in keratinocytes	^231^	
Dopamine	Inhibition of hair growth		Through the stimulation of catagen induction	^98^	
Serotonin	Involved in skin pigmentation		Modulation of melatonin	^16,223^	
Acetylcholine	Barrier function		Not reported	^16^	
Phenol & p-cresol	Impaired epidermal barrier function		Skin hydration reduction and disruption of keratinization	^171,232^	
Propionic acid	Promote skin homeostasis by reducing inflammation		Antimicrobial effects	^2^	
Sodium butyrate	Treat psoriasis and other hyperproliferative skin diseases		Modulation of several key cellular processes including differentiation, proliferation, and apoptosis.	^137^	
Galactoligosaccharides and fructooligosaccharides	Reduction of infant eczema and allergy		Through the stimulation of Tregs	^13,107^	
Polysaccharide A and retinoic acid	Suppress inflammation		Induction of accumulation of Tregs	^153^	
Saturated fats and higher amount of glycemic load	Development of acne		Impairment in nutrient signaling. SREBP-1 overexpression and increased sebum synthesis of fatty acids (e.g., free oleic acid) and triglycerides which promotes flourishing. *P. acnes* growth	^107^	
High-peptides and unsaturated omega-3 fatty acids	Act against hypersensitivity (allergies) and asthma		Through the development of Tregs	^13^	
High-fat and alcohol	Promote skin inflammation and oxidative stress. Impairment of colonic epithelial integrity and barrier function.		Increase of pro-inflammatory cytokines secretion	^122,123^	

### Diet, drugs and other consumed substances affect skin through gut microbiome

Several studies have related the diversity and pathogenicity of the gut microbiome to skin disorders, which can be significantly altered by long-term dietary patterns.^43,105–107^ Diet can affect the skin condition both positively and negatively through alteration of the gut microbiome, indicating that there is a relationship between the skin and the gut.^16^ Not only diet, but also many synthetic and natural products consumed by humans as drugs can provide direct and indirect evidence on the connection between gut microbiome and skin. Direct effects of antibiotics are perhaps the finest example of the link between gut and skin microbiota.^108^ Although the prime target of antibiotics use is the elimination of infectious pathogens, they can also be useful in treating noninfectious cutaneous diseases because of their anti-inflammatory and immunomodulatory properties.^109,110^ In contrast to antibiotics, prebiotics and probiotics invigorate the gut mirobiome.^111^ Prebiotics are typically known as nondigestible carbohydrates, which promote the flourishment of probiotic bacteria in the gut.^112,113^ On the other hand, probiotics influence the gut and skin according to nutritional status and medical conditions.^113^ They are the key players in balancing the gut microbiome, which eventually also regulates human health.^223^ The effects of diet, antibiotics, prebiotics, probiotics, and novel biologic drugs on the relationship of the gut microbiome with skin health are discussed below.

#### Breastmilk and formula

The gut microbiome of a newborn is highly dependent on diet. Breastfed infants have much higher levels of bacteria belonging to the class Actinobacteria.^114,115^ Breastfeeding influences gut microbiome diversity by increasing especially colonization of genera belonging to *Lactobacillus* and *Bifidobacterium*.^114–118^ Colonization of the gut by the bacterial class γ-Proteobacteria, which contains many proinflammatory species^118^ is often observed in formula-fed infants.^119^ Furthermore, babies fed with formula are more likely to be colonized by members of the phylum Bacteroides, and the opportunists *Escherichia coli* and *Clostridium difficile*.^13,120^ High quantities of oligosaccharides and various fatty acids present in breast milk positively influence the gut microbiome and its metabolites that can act against hypersensitivity (allergy) and asthma through stimulation of Tregs.^13^

#### High and low-fat diet

In the gut, a diet high in industrial trans-fatty acids increases the number of harmful microbes (such as *Desulfovibrionaceae* and *Proteobacteria*) while suppressing populations of advantageous microorganisms (e.g. members of Bacteroidetes, Lachnospiraceae, and Bacteroidales).^121^ Refined and hydrogenated oils (e.g., soybean, sunflower, safflower, canola, corn, and vegetable oils) can cause inflammation in the gut, which then manifests on the skin. Skin wound healing may also be delayed by a high-fat diet and alcohol, which exacerbate the inflammation of the skin and oxidative stress.^122^ High-fat diets are responsible for reducing gut microbial diversity and inducing the production of higher concentrations of lipopolysaccharides. This leads to a loss of colonic epithelial integrity and barrier function, a reduction in mucus layer thickness, and an increase in the release of pro-inflammatory cytokines, all of which further lead to systemic inflammation.^123^

#### Protein-rich diet

The gut microbiome can synthesize and subsequently export excessive dietary proteins and amino acids, resulting in the production of indoxyl sulfate (IS), trimethylamine N-oxide (TMAO), and p-cresyl sulfate-like toxins.^5^ These toxins are involved in several skin diseases, e.g., peripheral arthritis and psoriasis.^106,124^ In contrast, a high-collagen peptide diet, which contains high quantities of microbes, can protect the skin from aging and promote wound healing.^125^ The abundance of gut-commensal genera, such as *Lactobacillus* and *Bifidobacterium*, is increased with the ingestion of a diet containing whey and pea protein extracts, and the presence of pathogenic *Bacteroides fragilis* and *Clostridium perfringens* is simultaneously decreased. SCFA levels in the intestinal mucosa are increased with the consumption of pea proteins, which are critical for keeping the mucosal barrier intact.^106^

#### Dietary fiber

In general, a fiber-rich diet has a remarkably beneficial effect on the gut microbiome and is therefore well studied. Dietary fiber cannot be digested by the human body and is mainly metabolized by the microbiome in the colon.^105^ The metabolic process induces the growth of many bacterial groups. Eating foods rich in dietary fiber, especially whole grains, dramatically increases the populations of Bifidobacteria and the *Lactobacillus*/*Enterococcus* group.^105^ Foods containing complex dietary carbohydrates can be converted into SCFAs, including propionate, acetate, and butyrate through fermentation by the gut microbiome.^2,5,105,120^ Gut commensal microorganisms influence mucosal immunity through the development of Tregs within the colon, which is mediated through SCFAs.^120^ By increasing epithelial barrier function and inducing various anti-inflammatory effects, the SCFAs strengthen the gut’s function and integrity,^5^ modulate respiratory diseases,^13^ prevent the development of inflammatory disorders, e.g., allergy, arthritis, and colitis,^2^ regulate the metabolism of lipids and glucose,^5^ and inhibit the buildup of potentially harmful metabolic by-products, such as D-lactate.^41^ SCFAs play a role in the expression of Foxp3 (Forkhead box protein P3) that helps in regulating the development and function of Tregs, thus improving the regulatory T cell function.^126,127^ The anti-inflammatory effects by SCFAs are aided by G-protein coupled receptor 43, TGF-β, and/or IL-10, in addition to Tregs.^128^ On the skin, the anti-inflammatory actions could be mediated through resident Tregs, which become less abundant in certain inflammatory dermatoses.^120^ SCFAs derived from fiber through the gut can also influence the prevalence of certain skin microbial groups, which subsequently affect cutaneous immune defense mechanisms. By preventing the growth of harmful bacteria on the skin and lowering inflammation, the skin microbiome can collaborate with the immune system to promote skin homeostasis.^26^ For example, propionic acid, which is formed during the fermentation of dietary fiber by the *Propionibacterium* genus, is antibacterial and can kill the most prevalent community-acquired methicillin-resistant *Staphylococcus aureus* strains USA300.^2^

#### Antibiotics

One of the most well-studied effects on the gut microbiome is that of antibiotics. They have the potential to change the composition and function of the gut microbiome by killing or inhibiting the growth of specific microbial groups and by changing the molecular patterns associated with the microbiome. The effect of antimicrobials on the gut microbiome leads to dysbiosis,^108^ and consequently, skin abnormalities may occur. For example, oral administration of vancomycin for the treatment of skin wounds reduced bacterial diversity and regenerating gene III gamma (RegIIIγ) expression, potentially delaying wound-healing repair mechanisms.^226^ Antibiotic use, over time, leads to spread of antibacterial resistance and a decline in non-target bacterial populations, which can result in flourishment of pathogenic bacteria or fungi.^111,129^ For example, the use of a set of antimicrobials, levofloxacin and moxifloxacin, is associated with significantly increased numbers of *Candida* species in the human gut.^130^ The yeast *Candida* can colonize the small intestine and manifest as skin redness, which in turn accelerates aging.^131^

#### Prebiotics

International Scientific Association for Probiotics and Prebiotics (ISAPP) declares that the substrates that upon utilization by microorganisms provide useful properties to the host are prebiotics.^132^ They play a significant role in enhancing the number of gut microbes and their function.^133,134^ Prebiotics, such as fructooligosaccharides, galactooligosaccharides, inulin, polydextrose, lactulose, sorbitol, and xylitol are a promising group of compounds that modulate the gut microbiome and can also provide skin benefits.^135^ The prebiotics, galactooligosaccharides (GOS), are one of the major components of breast milk that can be fermented into SCFAs by members in the genus *Bifidobacterium*. This was shown by the number of human milk oligosaccharides (HMOs) decreasing in feces along with the increased presence of *Bifidobacterium* genera. HMO utilization by *Bifidobacterium* spp. can be considered as a prebiotic impact on the infant gut microbiome. Similarly, the human milk fatty acid palmitate plays a prebiotic effect on the infant gut microbiome by positively influencing the abundance of *Bifidobacterium* spp. and *Lactobacillus* spp.^43^

The effect of prebiotics on the skin condition is also obvious. For example, a *Lactobacillus* extract helps to reduce the size of acne lesions as well as inflammation by reducing skin erythema, improving skin barrier function and lowering the microbial counts on skin.^136^ Furthermore, GOSs have been used in the treatment of photoaging diseases for decades.^137^ In a study, the combination of GOS and *Bifidobacterium* reduced trans-epidermal water loss (TEWL or TWL) and prevented skin erythema.^137,138^ Atopic dermatitis and eczema have also been treated with GOS.^139^ Another prebiotic (carbohydrate) derived metabolite, sodium butyrate,^140^ is commonly used to treat hyperproliferative skin diseases (including psoriasis) through its modulation of key cellular processes, such as differentiation, proliferation, and apoptosis.^137^ Sodium butyrate is a compound derived from the gut microbiome that commonly affects the cell cycle, protease enzymes, and tumor growth factors (TGF-β). Apoptosis in keratinocyte (HaCaT) cells can be induced by sodium butyrate through up-regulation of the Fas receptor (death receptor) with the concomitant triggering of caspases 8 and 3. Another effect is an induced expression of p52 and TGF-β, suggesting a connection with cell proliferation and terminal differentiation.^137^ Differentiation of keratinocytes can also be enhanced by the joint action of sodium butyrate and PD153035 (an epidermal growth factor receptor inhibitor).^141^

#### Probiotics

Probiotics are living organisms that, when consumed in sufficient proportions, confer health benefits. They can be formulated, for example, as food, drugs, and dietary supplements.^6,113,142^ Probiotics can prevent gut colonization by pathogens and support anti-inflammatory responses by producing metabolites with anti-inflammatory properties. The most common probiotic microbes currently in use belong to the genera *Bacillus, Bifidobacterium, Enterococcus, Escherichia, Lactobacillus, Saccharomyces*, and *Streptococcus*.^143,144^ Several beneficial effects of probiotic consumption have been demonstrated on many dermatological conditions, thus proving the existence of the gut-skin axis. For example, skin sensitivity and significant restoration of skin barrier function were reported in individuals after daily oral administration of *Lactobacillus paracasei*.^145^ In a study conducted on mice, addition of *Lactobacillus reuteri* to drinking water resulted in improved epidermal thickness, increased folliculogenesis, lower skin pH, and enhanced production of sebum-producing epithelial cells. Accordingly, mice provided with the supplemented water had shinier and thicker fur than those that did not receive the probiotic.^2,3^ In another experiment, administration of *Lactobacillus johnsonii* in mice exhibited a therapeutic effect in restoring skin damage after exposure to UV radiation.^15^ The beneficial role of probiotics in preventing dermatological diseases has also been shown. For example, administration of *E. coli* strain Nissle improved skin health in patients with acne vulgaris.^15,146^ Orally administered doxycycline and probiotics were effective in a study in patients suffering from rosacea.^135^ In another study, the probability of developing atopic dermatitis was decreased throughout the postnatal period in mice by *Lactobacillus sakei* WIKIM30 consumption. The mice that had probiotics exhibited altered gut microbial composition along with improved skin lesions due to induction of Tregs. The risk of atopic dermatitis development was also found considerably lower in children who took probiotic supplements in the post-neonatal period of life.^15^

#### Novel biologic drugs

‘Novel biologics’ refers to substances generated from products of biological entities and may become therapeutic for dermatological conditions.^147^ A few immune-based diseases are now being treated with novel biologic drugs by therapeutic manipulation. Some therapeutic biologicals authorized by the European Medicines Agency are Adalimumab (Humira), Infliximab (Remicade), Rituximab (MabThera), and Etanercept (Enbrel). All of these drugs have a direct involvement with skin and gastrointestinal tract.^148,149^ Among all intrinsic and extrinsic factors, drugs are indicated as the major one that can remarkably interrupt gut microbial ecology. Gut microbial composition may be affected by drugs in two common ways. First, drugs can influence the translocation of microbes from other body parts to the intestine, and secondly, they can affect the local bacterial growth directly.^150^ Biologics such as Etanercept and Infliximab, which are used to treat psoriasis, may affect some microbial species, e.g. *Clostridium citroniae*, *Collinsella aerofaciens*, *Dorea formicigenerans*, *Parabacteroides distasonis*, *Prevotella copri*, and *Ruminococcus gnavus*.^149,151^

Technological advancements have opened up new possibilities for the selection and manufacture of monoclonal antibodies that target specific diseases.^149^ For example, the only monoclonal antibody authorized for individuals suffering from chronic spontaneous urticaria is omalizumab. These novel biologics are regarded as an efficient and safe treatment, but their availability and high cost severely impair their usefulness.^152^

### The link between gut and skin disease

Although a healthy gut is essential for host health, gut microbiome overgrowth and changes in diversity can result in a skin disease. Specific metabolic byproducts of gut microbes can directly influence normal physiology and disease processes,^5^ as discussed in previous paragraphs. Factors that influence the diversity and composition of the gut microbial community can be grouped into categories, namely non-host factors (such as environmental determinants), host factors (such as pancreatic enzymes, bile acids, pH), and bacterial factors (such as microbial enzymes, adhesive ability).^225^ Gut dysbiosis^153^ negatively impacts skin health and is considered to contain biomarkers such as free phenol, p-cresol, and aromatic amino acid derivatives produced by a disturbed gut.^3^ Dysbiosis in the gut contributes to the three common skin disorders: psoriasis, atopic dermatitis, and acne.^2^ There are also reports on the association of gut dysbiosis with some less common but potentially more serious diseases, such as rosacea, alopecia areata, hidradenitis suppurativa,^15^ erythema nodosum, and pyoderma gangrenosum.^135^ Furthermore, epidemiological studies and clinical trials suggest that modulation of the gut microbiome may influence susceptibility to allergic diseases and asthma.^154^

The skin plays a crucial part in maintaining homeostasis of the body by performing several important functions, such as regulation of water balance and temperature control. For these functions to be performed, the skin must undergo a renewal and turnover process, known as skin regeneration. Once epidermal cells differentiate from stem cells, epidermal cells undergo a differentiation process called keratinization, which is regulated by specific transcriptional processes. The gut microbiome influences the signaling processes that maintain epidermal differentiation and thus affect skin homeostasis.^155^ Several studies provided more information regarding a correlation between the gut microbiome and skin health. For example, the genetic material of gut bacteria has been identified in plasma samples of patients with psoriasis. In the study of 54 patients and 27 healthy controls, bacterial DNA was found in 16 out of 54 plaque psoriasis patients and was not detected in any controls. Furthermore, those patients with psoriasis were also detected with an increase in systemic inflammatory response markers (SIRs) such as IFN-γ, IL-1β, IL-6, IL-12, and TNF compared with the healthy control. Sequencing of the bacterial DNA identified the same type of organisms commonly found in the gut flora, pointing toward a possible link between the gut microbiome with psoriasis.^3,5,156^ Patients with Crohn’s disease are also mostly encountered with psoriasis as a comorbidity.^107,155^ Such findings suggest a correlation between altered gut microbiome and skin condition.^2^ Table 2 represents several human clinical studies that have used gut microbial interventions for human skin diseases. Next, we will take a closer look at the specific diseases where gut microbiome affects the development of the condition.

**Table 2.** Human clinical studies that have used gut microbial interventions for human skin diseases.

Participants	Intervention	Key findings	References
43 children (aged 4–17 years)	*Bifidobacterium animalis* subsp. *lactis* CECT 8145, *Lactobacillus casei* CECT 9104, and *Bifidobacterium longum* CECT 7347 (mixture) for 12 weeks.	Reducing the severity of AD according to the SCORAD index	^233^
50 children (aged 4 to 17 years)	*Bifidobacterium lactis* CECT8145, *Bifidobacterium longum* CECT 7347, and *Lactobacillus casei* CECT 9104 (mixture) for 12 weeks	Reducing the severity of AD according to the SCORAD index	^234^
31 patients (aged 18–75 years)	*Lactobacillus johnsonii* NCC 533, twice daily for 3 weeks	Reducing the severity of AD according to the SCORAD index	^235^
20 Caucasian adult	*Lactobacillus rhamnosus* for 12 weeks	Reducing the acne severity	^236^
101 Japanese female students (aged 18–23 years)	*B. breve* strain Yakult (YIT 12272), *Lactococcus lactis* YIT 2027, and *Streptococcus thermophilus* YIT 2021 (mixture) for 4 weeks	Enhancing stool consistency, defecation frequency, and feces quantity to maintain healthy skin	^237^
110 volunteers (aged 41 and 59 years)	*Lactobacillus plantarum* HY7714 for 12 weeks	Enhancing skin hydration, reduction of wrinkle depth, and overall skin shine and elasticity	^238^
166 pregnant women	*Bifidobacterium breve* M-16 V and *Bifidobacterium longum* BB536 for 4 weeks	Reducing the risk of newborns eczema	^239^
45 females (aged 18 to 35 years)	*Lactobacillus acidophilus*, *Lactobacillus delbrueckii*, and *Bifidobacterium bifidum* (mixture) for 12 weeks	Improving the total lesion for acne patients	^240^
26 male and female (aged 18–60 years)	*Bifidobacterium infantis 35624* for 6‒8 weeks	Improving the CRP levels in psoriasis patients	^170^
30 women (aged 30–48 years)	*L. lactis strain* H61 (60 mg), daily for 8 weeks	Improving hair follicles and skin hydration	^241^
A woman (aged 47 years)	*Lactobacillus sporogenes* and biotin (mixture)	Reducing the risk of psoriasis	^242^
20 children (aged 4–15 years)	*L. acidophilus* L-92 strain for 8 weeks	Effective as Serum Markers of Atopic Dermatitis in Children	^243^
56 Patients (aged 18 to 30 years)	*L. bulgaricus* and *Streptococcus thermophilus* for 12 weeks	Reducing the severity of acne vulgaris by selectively lowering TGs in skin surface lipids.	^244^
66 female volunteers (aged 35–45 years)	*Bifidobacterium longum*, twice daily for 2 months	Improving skin hydration and physical aggression	^245^
70 patients (12 years of age or older)	*Enterococcus faecalis* SL-5 for 8 weeks	Improving the severity of acne	^246^
474 patients	*Lactobacillus rhamnosus* for 35 weeks	Reducing the risk of eczema	^247^
59 patients	*Lactobacillus rhamnosus* and *Bifidobacterium* *lactis* for 12 weeks	Reducing the severity of AD according to the SCORAD index	^248^
56 children (aged 6–18 months)	*Lactobacillus fermentum* VRI-033 PCC for 8 weeks	Reducing the severity of AD according to the SCORAD index	^249^
41 children (aged 1–13 years)	*Lactobacillus rhamnosus* 19070*–2* and *Lactobacillus reuteri* DSM 12246 for 6 weeks	Reducing the severity of atopic eczema	^250^
159 pregnant women	*Lactobacillus rhamnosus* strain GG for 4 weeks	Reducing the severity of atopic eczema	^251^
159 patients	*Lactobacillus* GG for 4 weeks	Reducing the severity of atopic eczema in children	^252^
27 infants	*Bifidobacterium lactis* Bb-12 or *Lactobacillus* strain GG (ATCC 53103)	Reducing the severity of atopic eczema	^253^

### Psoriasis

Psoriasis is a systemic autoimmune condition associated with improper activation of immune-mediated pathways that drive the immune cells to attack self-skin cells, subsequently resulting in increased levels of pro-inflammatory cytokines. Inflammatory bowel disease (IBD), a second known comorbidity of psoriasis, is associated with the gut microbiome through development of pro-inflammatory Th17 cells.^5,157^ A reduction in the abundance of potentially beneficial microbes in psoriasis patients could alter balance of immune system, thus affecting skin health.^158,159^ Specifically, psoriasis patients have reduced numbers of Bacteroidetes (including the *Bacteroides* genus), Proteobacteria, and Actinobacteria along with relatively low amounts of *Akkermansia muciniphila*^15,160,161^ and a lower prevalence of the phylum Firmicutes.^162^ A reduced abundance of *Akkermansia* and *Ruminoccocus* was also observed in patients with psoriasis, psoriatic arthritis (PsA), and IBD.^159,160,163^

Biomarkers of a damaged intestinal barrier, such as elevated levels of claudin-3 and intestinal fatty acid-binding protein, have been observed among psoriasis patients.^15^ Furthermore, microbial production of TMAO upon utilization of choline can lead to psoriasis.^106,124^ Several studies have shown that *Bacteroides* produces polysaccharide A, activates Tregs and promotes the anti-inflammatory response.^158,164–166^ Thus, a remarkable reduction in *Bacteroides* count in psoriasis could lead to immune alteration and pro-inflammatory response.^158^ As the natural colonizers of the mucin layer in the human gut, *Akkermansia muciniphila* and *Ruminoccocus* prevent pathogen colonization by competitive inhibition.^157,159,167,168^ Moreover, *Bacteroides, Akkermansia muciniphila* and *Ruminoccocus* produce SCFAs.^126^ Therefore, the reduction of these beneficial microbes results in the impairment of the gut barrier function, promoting bacterial translocation from the gut into the systemic circulation in psoriasis patients.^126,156,166^

Oral administration of antibiotics, prebiotics, probiotics, and newly developed fecal transplantation are promising therapeutic methods for psoriasis.^169^ Probiotics have been shown to improve the disease course of psoriasis by increasing TNF-α (tumor necrosis factor alpha) production by epithelial cells, by improving the barrier function, and by regulating the NF-kβ (Nuclear factor kappa B) pathway that affects the development of psoriasis pathogenesis.^169^ A study by Groeger and colleagues in 2013 found that *Bifidobacterium infantis* 35624 supplementation significantly decreased plasma levels of TNF-α, IL-6, and C-reactive protein (CRP) (the markers of inflammation in the body) among psoriasis sufferers.^170^ Although studies have shown that probiotic treatments lead to a significant change in skin inflammation, there is still no evidence to support their use in the treatment of psoriasis. Therefore, further research is needed in this field.^171^

### Atopic dermatitis (eczema)

Several investigations on the role of gut microbiome in the pathogenesis of atopic dermatitis (AD) in infants and children suggest that infants with limited heterogeneity in the gut microbiome develop AD later in life.^5,172–175^ This is contradicting to other results, which suggest that gut microbial diversity facilitates AD. For example, a lower abundance of *Bifidobacterium* in the gut was observed in patients with AD compared to healthy controls.^15,176,177^ However, alteration of gut microbial composition alone cannot promote the development of AD. Interaction of specific microbes with the immune system, together with other external factors, such as diet, could better explain the pathogenesis of AD. The immunopathology behind AD prognosis is linked with an imbalance of Th1 and Th2. The effector skin-resident dendritic cells of AD patients travel to the local lymph node where they activate the naïve T lymphocytes and induce their differentiation into effector Th2 subtype of effector T lymphocytes.^223^ Once activated and recruited back to the skin, Th2s substantially produce inflammatory cytokines, such as IL-4, IL-5, and IL-13, which ultimately causes enhanced production of IgE, commonly found in AD patient.^178,223^

Two metagenomic studies conducted in South Korea analyzed fecal samples of patients with AD and revealed a decreased abundance in *Faecalibacterium prausnitzii* along with a significant reduction in SCFA production when compared with healthy controls.^2,179,180^ According to a study, dysbiosis of *F. prausnitzii* in patients with AD was associated with an increased expression of a variety of nutrients. These nutrients, such as the mucin components GalNAc and L-fucose, are released from damaged gut epithelium, indicating a leaky gut and dysregulation of inflammation of the gut epithelium. Increased gut permeability was identified due to damaged gut epithelium, allowing various toxins, food residues, and pathogens to access blood circulation. The metabolites and toxins ultimately paved their way to the skin and induced Th2-type immune responses by releasing pro-inflammatory cytokines, which underlies the prognosis of AD.^180^

Zheng et al.^181^ demonstrated that an increased abundance of *Akkermansia muciniphila* in infants with AD is associated with intestinal barrier dysfunction and skin lesion deterioration.^15,181^ In addition to, or potentially mediated by the gut microbiome, diet may also impact AD. For example, a gluten-containing diet can impair the intestinal barrier, causing leaky gut, and gluten sensitivity is associated with gut dysbiosis.^135,182,183^ According to a recent study, probiotic lactobacilli and enterococci of human origin can help to improve gut microbiome dysbiosis, and hence could be used as a treatment for disorders involving low SCFA synthesis in the gut.^184^ Furthermore, Fecal microbiota transplant (FMT) could be a potential therapeutic option for AD.^185^ However, research-oriented studies in this section are required to gain a better insight for future therapeutic interventions.

### Acne vulgaris

Acne vulgaris is identified by inflammatory skin lesions (papules and pustules), non‐inflammatory comedones, or a combination of the two.^15,135^ Several studies have indicated a correlation of gut dysbiosis with acne vulgaris. A recent investigation revealed a significant reduction in the prevalence of *Actinobacteria, Bifidobacterium, Butyricicoccus, Coprobacillus*, and *Lactobacillus* species along with an increased abundance of Proteobacteria in persons with acne vulgaris.^15^ According to a hypothesis, sterol regulatory element-binding protein 1 (SREBP-1), sebum fatty acid, and sebum triglyceride become stimulated by nutrient signaling disruption and lead to flourishment of *Propionibacterium acnes*.^186^ In addition to the gut microbiome, various metabolic pathways also influence the pathophysiology of acne vulgaris, such as the mTOR pathway, which becomes activated by high glycemic load. The high glycemic load is the sole contributor to increased insulin/insulin-like growth factor (IGF-1) signaling, enhancing the cytoplasmic expression of FoxO1 (Forkhead box transcription factor O1). FoxO1 then stimulates the mammalian target of rapamycin complex 1 (mTORC1), which ultimately leads to acne development.^2^

Besides the mTOR pathway, high-fat diet has an influence on acne development.^187^ Guo et al. found that there is a lower release rate of AMPs in the small intestine of mice due to high-fat diet, which promotes growth of Firmicutes than Bacteroidetes, resulting in dysbiosis and alteration of serological cytokine levels that promote inflammation.^188^ Several acne patients have also been found to develop Hyperchlorhydria (low level of stomach acid). Low acidity levels allow colonic bacteria to migrate to the distal region of the small intestine, creating conditions for intestinal dysbiosis and growth of small intestinal bacteria, which can increase intestinal permeability and promote inflammation of the skin.^189^

Noureldein & Eid^190^ demonstrated that the inhibition of mTORC1 together with changes in the gut microbiome improve the level of glucose tolerance and reduce hyperinsulinemia. Supporting this hypothesis, a reduced level of acne lesion was found in patients supplied with a low glycemic load diet for 12 weeks.^191^ Also, probiotics may reduce acne inflammation by reducing levels of inflammatory cytokines, by increasing CD8 cell recruitment, by suppressing IL−1α, and by activating Tregs.^112,192^

### Rosacea

Rosacea is a common dermatological condition that is characterized by pustules, persistent erythema, excessive fibrous tissue proliferation, papules, telangiectasia, and periocular changes. The coexistence of several gastrointestinal comorbidities with rosacea has been observed, which connects the condition with altered gut microbiome.^15,107,161^ Moreover, *H. pylori* infection, IBD, and small intestinal bacterial overgrowth (SIBO) are potentially associated with the pathogenesis of rosacea.^15,135,161^ The crosstalk between the immune system and the gut-associated diseases further demonstrates the link between gut and rosacea. According to reports, the role of *H. pylori* in rosacea pathogenesis is associated with several immune-pathological and inflammatory mediators and toxic factors.^193–195^ For example, *H. pylori* can substantially increase ROS generation (Reactive oxygen species), which has an inflammatory effect on the gut. Among the ROS, NO (nitric oxide) causes inflammation of the gut mucosa and alters the skin physiological processes, including vasodilation, inflammation and immunomodulation, ultimately resulting in the clinical manifestations associated with rosacea.^194^ Another mechanism justifying the role of *H. pylori* in rosacea development is the production of a cytotoxin, which induces the production of pro-inflammatory cytokines such as TNF-α and IL-8. As a result of these inflammatory cytokine releases, inflammation of gastric mucosa and clinical manifestation of rosacea are seen.^193,194^

The eradication of *H. pylori* reduces rosacea symptoms as well as associated gastrointestinal issues.^196^ *Bacillus subtilis*-3 is effective against *H. pylori*, allowing spore colonization of the gastrointestinal system and subsequent microbiome modification by permitting passage over the gastric barrier.^197^ Many studies have shown that probiotics can be used to treat chronic inflammatory rosacea, which could be used as an additional therapy in patients.^198,199^

### Alopecia areata (AA)

AA, or spot baldness, is an autoimmune disorder in which the hair-loss pattern is in either a limited or all areas of the body. AA has no age restriction.^15^ AA is an auto-immune disease where the auto-reactive T lymphocyte interacts with a follicular auto-antigen presented by perifollicular or follicular cell. Their interaction gives rise to activation and induction of the T Lymphocytes, especially Th1, to produce pro-inflammatory cytokines (such as IFN-γ). IFN-γ disrupts the anagen growth phase, ultimately resulting in hair loss and other manifestations of AA.^200,201^

When compared with a healthy population, a high proportion of persons with AA also exhibit ulcerative colitis, indicating an association of the gut with AA.^15,200,202^ The driving force behind this association is accumulation of auto-reactive T lymphocytes that gain tolerance against apoptotic cell death, causing prolonged chronic inflammation and hair loss due to inflammatory cytokine (IFN-γ and IL-2) production by the autoreactive Th1 cells.^200^

FMT is thought to be a safe and effective way to reestablish a healthy gut microbial ecosystem by delivering beneficial bacteria and nutrients directly or indirectly.^203^ FMT could possibly be used to treat alopecia areata by restoring the homeostasis of the gut flora.^204^

### Hidradenitis suppurativa

HS is a chronic disorder of the skin characterized by inflammation of hair follicles in intertriginous areas.^15^ The mechanism underlying this chronic disorder needs to be deeply researched, as very little evidence and studies are available for inferring correlation with the disease pathogenesis and gut microbiome. Although the pathogenesis of HS is not yet clear, follicular occlusion syndrome, disturbance of the inflammatory cytokines, such as TNF-α, IL-1ß and IL-17, and fluctuation in the microbial composition are known to be crucial.^205–210^ The possible linkage between HS pathology and altered gut microbiome is supported by some features that are found in both HS and acne vulgaris, such as the coexistence of IBD and metabolic syndrome, and a negative association with a high-sugar diet.^15,211,212^ IBD was observed at high frequencies in patients with HS when compared with healthy controls. Persons with HS also had a significant reduction in *Faecalibacterium prausnitzii* and increased abundance of *E. coli* in their gut.^15,213,214^

Gut microbial dysbiosis can potentially induce HS by influencing skin microbes through mediating systemic inflammatory pathways.^2,215^ According to an interesting hypothesis, colonic dysbiosis resulting from high-fat diet results in inflammatory cytokine (e.g., TNF-α, IL-1β, and IL-17) elevation, and ultimately in formation of HS lesion by enhancing matrix metalloproteinase levels.^215^ Probiotics are considered an effective option in treating HS for restoring a healthy cutaneous microbiome, as they are capable of replenishing the abundance of *Cutibacterium* spp., *Corynebacterium*, and *Staphylococcus* within the communities.^216^ Avoiding a high-fat diet can be a great secondary preventive measure for HS.^217^ However, finding the potential primary preventive measure (e.g., microbial marker) for HS is still under rigorous research.

## Conclusion and future prospective

Understanding the relationship between gut and skin microbial communities is an emerging area of research. A number of findings we have summarized here provides sufficient evidence for the existence of a skin–gut axis. Although limited research is available on the pathobiological drivers on the gut-skin axis in disease, our review has provided a brief understanding on the linkage between the gut microbiome with skin diseases and indicated direction of potential future studies. Whereas specific skin diseases have been connected with gut health and the balance of microbiome within the gut, the exact mechanisms of influencing skin by intestinal microorganisms are to be elucidated. Besides acting through the immune system, catabolic products of diet and microbial compounds can impact the gut epithelium by altering gut physiology, leading to a variety of secretory products that circulate throughout the body and enter the skin. Skin commensal microbiome can be affected by the bioactive compounds, such as neurotransmitters, hormones, and SCFAs, which are the end-products of gut microbial metabolism. Ingested compounds and chemicals can thus have an immediate impact on the skin appearance and activity. However, the mechanisms behind this interaction are multifactorial and currently mainly based on theory. Further studies that can detail the fate of specific compounds (e.g., by labeling), being transferred from the intestine to the skin and their mechanism of action on skin cells and/or microbiome would be needed to positively prove such connection. For example, metabolites, such as ceramide,^218–227^ have been radioactively labeled prior to oral consumption and tracked on the skin of mice. Similar labeling studies on microbial products, such as SCFAs, would provide strong evidence toward direct effects of gut microbiome on the skin.

Another line of research having many promising prospects on the gut-skin axis are dietary supplements promoting health of gut microbiome, including pre- and probiotics. This research area has been massively studied in the past due to many beneficial health effects of fermented foods. In our review, we have listed several connections of probiotics with the skin condition, for example, *Lactobacillus reuteri*, which improves epidermal thickness and increased folliculogenesis after ingestion by mice.^3^ Further studies linking probiotics with skin would likely provide new significant knowledge on the importance of specific members of the gut microbial community in skin health. Linking together studies on probiotics and tracking of specific microbial products from gut to the skin could reveal exciting new information on the gut-skin axis in the future.

## Abbreviations

AA: Alopecia areata
AD: Atopic dermatitis
AhR: Aryl hydrocarbon receptor
AMP: Antimicrobial peptides
COL1A1: Collagen type 1 alpha 1
COL1A2: Collagen type 1 alpha 2
CRP: C-reactive protein
CX3CR1: C-X3-C Motif Chemokine Receptor 1
DC: Dendritic cell
FoxO1: Forkhead box transcription factor O1
Foxp3: Forkhead box protein P3
FMT: Fecal microbiota transplant
GalNAc: N-Acetylgalactosamine
GABA: Gamma-aminobutyric acid
GALTs: Gut-associated lymphoid tissues
GI: Gastrointestinal
GM-CSF: Granulocyte-macrophage colony-stimulating factor
GOS: Galacto-oligosaccharides
HS: Hidradenitis suppurativa
HMO: Human milk oligosaccharide
IFN: Interferon
IBD: Inflammatory bowel disease
IEC: Intestinal epithelial cell
IgA: Immunoglobulin A
IgE: Immunoglobulin E
IGF: Insulin-like growth factor
IMID: Immune-mediated inflammatory diseases
IL-10: Interleukin 10
ILCs: Innate lymphoid cells
ILFs: Isolated lymphoid follicles
IL: Interleukin
IS: Indoxyl sulfate
ISAPP: International Scientific Association for Probiotics and Prebiotics
MAIT: Mucosal-associated invariant T cells
MALTs: Mucosa-associated lymphoid tissues
LTi: Lymphoid tissue inducer
MHC II: Major histocompatibility complex II
mLN: Mediastinal lymph node
MMP: Matrix metalloproteinase
MR1: Major histocompatibility complex class I-related gene protein
mTOR: Mammalian target of rapamycin
mTORC1: Mammalian target of rapamycin complex 1
NF-kβ: Nuclear factor kappa B
NK: Natural Killer cells
NO: Nitric oxide
NLR: NOD like receptor
NOD: Nucleotide oligomerization domain
PRRs: Pattern-recognition receptors
PAMPs: Pathogen-associated molecular patterns
RegIII: Regenerating gene III
RIG: Retinoic acid-inducible gene I
RLR: RIG-like receptor
ROS: Reactive oxygen species
SIBO: Small intestinal bacterial overgrowth
SCFA: Short chain fatty acid
SREBP-1: Sterol regulatory element-binding protein 1
SIRs: Systemic inflammatory response markers
Tregs: T regulatory cells
TEWL or TWL: Trans-epidermal water loss
TLR: Toll like receptor
TMAO: Trimethylamine N-oxide
TGF-β: Tumor growth factor beta
TNF-α: Tumor necrosis factor alpha
TSLP: Thymic stromal lymphopoietin
Th1: T helper cell type 1
Th2: T helper cell type 2
